# How Urban Parks Offer Opportunities for Physical Activity in Dublin, Ireland

**DOI:** 10.3390/ijerph15040815

**Published:** 2018-04-21

**Authors:** Eve Burrows, Margaret O’Mahony, Dermot Geraghty

**Affiliations:** 1 Department of Mechanical and Manufacturing Engineering, Trinity College Dublin, the University of Dublin, Dublin 2, Ireland; burrowse@tcd.ie (E.B.); tgerghty@tcd.ie (D.G.); 2 Trinity Centre for Transport Research, Department of Civil, Structural & Environmental Engineering, Trinity College Dublin, the University of Dublin, Dublin 2, Ireland

**Keywords:** parks, visit frequency, physical activity

## Abstract

Parks are an important part of the urban fabric of cities. They offer people the opportunity to connect with nature, engage in physical activity, find a haven away from the city noise, or spend time alone or with family and friends. This study examines the relative importance of park and park visit characteristics for 865 survey participants in Dublin, Ireland. The data is analyzed using a multinomial logistic regression model which can distinguish the relative importance of attributes. The model results demonstrate an improvement over proportional by chance accuracy, indicating that the model is useful. The results suggest that when and why individuals go to the park along with the proximity of their residence to the park influence visit frequency more than their age and gender and more than their impression of the sound levels in the park. The contribution of the results, in terms of their potential usefulness to planners, suggest that the priority should be on the provision of park space close to residential areas, so that individuals can engage in activities such as walking and relaxation, and that the quality of that space, in the context of noise levels at least, is less important.

## 1. Introduction

In busy cities, parks offer people an opportunity for respite from noise, traffic, crowded streets, and the intensity of urban living. Godbey [1] recommended the examination of the role and relative influence of park characteristics on facilitating or limiting park use. The study presented here responds to that call by examining the factors that influence the frequency of park visitation. As a background, the review of previous research presented here focuses on three main topics: parks and the role they play in cities, their influence in promoting physical activity, and finally the role that location and other physical characteristics of the parks play in influencing park use.

Urban parks have been found to be important for city sustainability, and their design should take into account the recreational requirements of different age groups [2]. In Berlin, most sub-districts residents have access to sufficient urban green space in their vicinity, using the threshold value of 6 m^2^ per person [3]. In another German study, no inequalities in an analysis of distance to urban green space was found [4]. Other recent work examined the factors that influence the value of an urban park within a medium-sized French conurbation [5]. Frequency and age were found to positively influence the probability of willingness to pay to enjoy a park. Madureira et al. [6] examined the beliefs of urban residents concerning green space benefits in four cities in France and Portugal. Green spaces were not equally valued, but their importance for personal health and wellbeing and to facilitate contact with nature were noted by the residents of all four urban areas.

Other interesting research examined site configuration, microclimates, and users’ perceptions of green open space in high-density Asian cities [7]. The level of space enclosure and the greenery density are significantly associated with outdoor microclimate conditions and use behaviors such as visit patterns, sensation, and healing [7]. Social connection was the most highly rated factor by elderly people questioned in surveys across a number of parks in three urban renewal districts in Hong Kong [8].

Moving to the second background review topic which focuses on how parks influence the promotion of physical activity, the importance of urban parks for human well-being through recreational activities has been noted by some [4,9], whereas others found that green space is not a necessity for physical activity [6]. When urban green spaces were introduced to a disadvantaged area in Copenhagen, the time adolescents spent on physical activity in the district increased [10]. Research in Malaysia [11] found that people visited an urban park to get fresh air, to reduce stress, and to exercise. Those individuals with very good access to large, attractive, public open spaces were 50% more likely to achieve high levels of walking [12], and, in another study, the majority of park users engaged in moderate to vigorous physical activity [13]. Many benefits can be obtained by users of parks, but physical health benefits were found to be the main ones [14].

The third topic considered here in background review, focuses on previous research that examined the influence of different factors on park use. Earlier work has considered the proximity of parks to an individual’s home, and a study by Apkinar [15] found that the nearer an urban green space was to an individual’s home, the higher their engagement in physical activity. However, Brown et al. [16] noted that the distance to a park was not a significant predictor of physical activity, but park size was correlated with physical activity. The opportunity to use parks, in terms of proximity to them, was less important than an individual’s motivation by their orientation toward nature [17]. People living within 20 min from a park in Thessaloniki were willing to contribute a significant amount of money to support the project [18]. The accessibility and use of peri-urban green space for inner-city dwellers was examined in another piece of research using a comparative study between two European cities, namely, Ljubljiana and Edinburgh [19]. In both cities, the perceptions of distance to travel were the major barrier to the frequent use of peri-urban green spaces.

Work conducted in Hong Kong found exercise and exposure to clean air topped the list of park visit purposes [20]. Veitch et al. [21] considered the park attributes that encourage park visitation among adolescents. Playground slides were considered the most important attribute, followed by an absence of rubbish/graffiti, the presence of swings, walking/cycling paths, and BMX tracks/skate bowls, in that order. Popular activities in parks were biking, sitting, and walking [22]. It was recommended by Godbey [1] that the key factors influencing physical activity in the transition of individuals from college to life after school needed to be identified.

Another park characteristic that may influence park usage is its soundscape. This ties in with the requirement in the Environmental Noise Directive for cities to preserve quiet areas in agglomerations [23]. A number of researchers have interviewed park users to survey perceived sound sources but have not looked at how noise influences park visit frequency. Brambilla et al. [24] noted that urban parks did not comply with the noise limit issued by Italian legislation on protected areas. Acoustic comfort evaluation plays an important role in users’ acceptability of the urban park environment [25].There is a close relationship between landscape and soundscape experiences and visual and functional aspects should be considered in creating better soundscapes in park design [26]. 

The literature review found that there is a dearth of research focusing on what influences park visitation among adolescents [21], and other studies have focused on adults aged between 25 and 54 years of age [5,27]. Young adults and what might influence them to use parks do not feature in the literature. This study responds to the calls by others [1,28] for further research to be done in assessing the importance of parks in urban life, their influence on physical activity levels and relaxation, and how particular cohorts of the population use and benefit from them.

The research presented here was conducted at the same time as the authorities in Dublin were designating quiet areas in line with the European Noise Directive [23]. All areas designated were parks. As part of this research, therefore, we chose to examine if park visit frequency was associated with the perception of sound levels in the parks visited. The aim was to supplement previous research on this topic in the literature, examples of which are mentioned here. A survey of 146 individuals in China found that there was a significant relationship between park visit frequency and individual sound perception [29]. Gozalo et al. [30] found that the absence of noise in urban green spaces can explain 71% of the overall satisfaction experienced in urban green spaces. Liu et al. [31] found that the perception of individual sounds most influenced park visit frequency, whereas the length of stay was associated with the overall soundscape perception.

The specific research question considered in this study was to identify the factors that most influence the frequency of park use, with a particular focus on young adults. Frequency of park visits was selected as the variable of interest because it was the variable over which individuals had most choice. The factors considered were divided into three groups: (1) social, demographic, and locational; (2) reasons why individuals visit a park, with a particular focus on physical activity; (3) perception of the park in terms of noise levels. Within the context of the research question, the research also sought to explore differences in outcomes from previous research. For example, some research has found the nearer the distance of an individual’s home to urban green space, the higher their engagement in physical activity [15,32,33], but others found that the distance to a park was not a significant predictor [16]. For the purposes of this work, a park is defined as a public green area with open access for all members of the public.

The first novel aspect of the work presented here is the focus on young adults and how they use parks. The influence of sound levels and the perception of how quiet a park is on park visit frequency is another novel aspect of the research presented here. Finally, further exploration of topics in which agreement has not been reached by other researchers, e.g., the influence of proximity to a park, will also be completed as part of the research. A multinomial logistic regression model was used to assess the relative importance of the three groups of factors on the dependent variable—frequency of park use—with the aim of demonstrating the importance of parks in urban design and the importance of their availability to support physical activity needs.

## 2. Materials and Methods

### 2.1. Survey Method

An online survey using SurveyMonkey [34] was designed to assess why individuals use urban parks, the extent that they use them, and to capture their impression of them. The survey was distributed electronically to academic and administrative staff and to undergraduate students of University of Dublin, Trinity College (TCD), Ireland, in 2015. Registered undergraduate students and staff at the time totalled 12,224 and 2624, respectively. Five hundred and eighty-five students and 280 staff responded to the survey; a response rate of 11% for staff and 5% of students was obtained. The responses were therefore heavily biased, as they only included people working and learning at university. While the response rates by population type were relatively low, the absolute number of responses of 865 was useful in the context of the topic under consideration. Ethics committee approval for the survey was sought and obtained from the TCD School of Engineering Ethics Committee before commencing with data collection, with the condition that the data be destroyed after analysis.

### 2.2. Questionnaire

In the survey, the respondents were asked to answer the questions in relation to any park they used. The individuals were asked which parks they visited or had visited previously and they could choose from the list of the parks specified in the survey or select a response of ‘other’ if they used alternative parks. The first part of the questionnaire included questions about gender and age. The next set of questions explored a number of variables relating to park visits. Whether the individuals visited the park alone or with others, the proximity of their residence to the park, the days of the week they typically visited the park, the typical duration of their stay, and the main reasons for visiting the park made up this section of the questionnaire. This was followed by two further questions. Firstly, their impression of the sound level in the park was sought, and, secondly, whether they considered that the park was valuable as a quiet area within their community. The actual wording of each question is presented in Table 1.

### 2.3. Data Analysis Method

A multinomial logistic regression (MNL) was developed with frequency of park visits as its dependent variable, and, as independent variables: gender, age, whether the individuals visited the park alone or with others, the proximity of their residence to the park, the days of the week they typically visited the park, the typical duration of their stay in the park, the main reasons for visiting the park, their impression of the sound level in the park, and whether they considered that the park was valuable as a quiet area within their community. The results are presented on the basis of three models: (1) park visit frequency of >3 times per week; (2) weekly park visit frequency; (3) monthly park visit frequency. Logistic regression can be used to predict the relationship between a dependent variable and a group of independent variables that are continuous or categorical. It can also rank the influence of the independent variables. The performance of the resulting model can be assessed by looking at the classification table, using the likelihood ratio test and the Nagelkerke statistic [35].

## 3. Results

At 62%, the majority of respondents were female, but this reflects the fact that the majority (58%) of undergraduate students at TCD are female. The age distribution also reflects the large student group with 55% in the age group being 18–24 years old. The second largest respondent group was the 35–49 age group (18%), followed by the 25–34 age group at 15%, and, lastly, 11% of the responses were from the >50 age group.

Most individuals (56%) visit the park with others, 30% visit it alone, and the remainder do both. In relation to how close they live to the park, most (57%) live within walking distance, 20% within a 10 min driving distance, and the remainder live further away. Most people visit the park in weekends (55%), 24% visit it on weekdays, and the remainder visit it on any day of the week. Fifty-one per cent typically spend an hour there, 34% spend less than 30 min, and 15% spend two or more hours. Thirty-four per cent said they go to the park to walk, 10% to run or jog, 35% to relax, and 16% to socialize.

Six per cent considered the park to be very quiet, 49% thought it was quiet, 40% considered the sound level to be adequate, and 5% found it to be noisy or very noisy. Thirty-nine per cent strongly agreed with the statement that the park was valuable as a quiet area, 43% somewhat agreed with that statement, 12% were neutral, and 6% somewhat or strongly disagreed.

The model fitting information from the MNL regression is presented in Table 2. The Nagelkerke R^2^ value of 0.4 represents effects with a relatively decent size [35]. The table shows that most of the variables included are significant at the 0.05 level, except for the sound level in the park variable and the respondents’ opinion about the value of the park as a quiet area. In relation to the chi-square values of the statistically significant variables, dropping the days of the week when visits to the park take place would result in the greatest loss of model fit, followed by the proximity of the respondents’ residence to the park, the main reason for using the park, whether they visit the park alone or with others, the duration of their stay, and their age, in that order. The classification accuracy rate was calculated to be 49.9% compared with 34% by chance alone accuracy, indicating the model’s usefulness.

The results of the logistic regression are presented in Table 3, in which the results from three models are shown, where the values in bold indicate statistical significance (*p*) at the <0.05 level. In the case of gender, only the weekly results were significant, and the odds ratio (OR) of 0.57 suggests that females were 43% less likely than males to visit a park on a weekly basis. This indicates a substantial difference between males and females for those who only visit the park weekly. However, as this result was not repeated for the other two frequencies corresponding to >3 times per week or monthly, which means that there was no difference between males and females in these cases, it is likely to be related to the purpose for which males visit the park on a weekly basis, e.g., team sports. For age, only in the case of 18–24-year-olds were the results significant in the case of monthly visits and they suggest that individuals in that age category were 2.1 times more likely to visit a park on a monthly basis than individuals in the >50 age group, which was used as the reference category in this case. As this result was not repeated for the other frequencies, it is difficult to highlight this result as demonstrating a major difference in the frequency of park visit behavior between the different age groups.

In the case of whether individuals visit the park alone or with others, the results for visiting with others were the only significant ones and they were significant across all three visit frequencies. With the OR values all around 0.3, this indicates that the individuals were 70% less likely to visit the park with others compared with the reference category, which in this case was visiting alone or with others. When examining the next variable in Table 3, proximity to the park, the results for all categories were significant across the three models. Individuals were eight times more likely to visit the park >3 times a week if they lived within walking distance (OR = 8.1), and over four times more likely if they lived within a 10 min drive (OR = 4.2) than those who lived further than a 10 min drive away. Similar indications were evident in the case of visiting the park weekly (OR = 7.5 and 3.66, respectively) and monthly (OR = 2.99 and 1.69, respectively). The statistical significance of these results and the strength of the OR values across all categories demonstrates the strong influence that proximity to a park has on park visit frequency. 

Those individuals visiting the park on any day of the week were more likely to visit the park more than three times per week (OR = 45) or weekly (OR = 2.7) compared with the individuals visiting the park only in weekends, which was the reference case here. When examining visits taking place on weekdays only, the OR was 9 for >3 times per week and 1.8 for weekly visits, again indicating much higher likelihoods of park visits happening on weekdays. The next variable considered was the duration of the respondents’ stay in the park. Durations under consideration were <30 min, 1 h and 2 or more hours, with the latter being the reference category. In this case, significance was only evident in the case of the second model (weekly visits). The OR of 0.23 indicates that those visiting the park weekly were 77% less likely to spend <30 min in it. This suggests that those visiting the park weekly stay for longer periods.

Those visiting the park >3 times per week were over three times most likely to be going there to walk (OR = 3.34), jog, or participate in sports (OR = 3.1) than to socialize, which was the reference category in this case. Significance was also evident for the same visit purposes in the case of the weekly model, indicating these respondents were over two times more likely to be going to the park to walk or jog (OR = 2.58 and 2.78, respectively). The final two variables, the first of which considered the perception of sound level in the park and the second how the individuals valued the park as a quiet area, showed no significance in any of the three models.

## 4. Discussion

The results demonstrate that the days of the week when individuals visit the park, the proximity of their residence to the park, and the reasons why they visit are the most important variables when predicting the frequency with which they visit parks. These variables are followed in importance by whether they visit the park alone or with others, the duration of their visits, the age, and the gender, in that order. How the respondents rated the sound level within the park and whether they considered it as a quiet area were considered not to be statistically significant in the analysis. In relation to the importance of the proximity of their residence to the park, the results support those previously reported [15,32,33,36]. Apkinar [15] found that the nearer an urban green space was to an individual’s home, the higher the individual's engagement in physical activity. While the research question examined here was not quite the same as that of Apkinar [15], 382 of the respondents said the main reason for visiting the park was for walking or jogging. By inference, this suggests that the park is important for them for engaging in that physical activity. Added to that, the robust results we obtained across all categories that suggested proximity to a park was a significant factor in influencing park visits, again complements the work of Apkinar [15]. Likewise, these results also align with those of Mowen et al. [36] who noted that physical activity and health as a result of park use frequency were related to the perceived walking distance to a park. The results also support the findings of another study [37], in that they show that individuals spend more time in the park if they visit it in weekends than on weekdays.

Walking, jogging, or taking part in sport were the main reasons for park visits, and these findings align with the results from Sreertheran [11], Lo et al. [20], Giles-Corti et al. [12], and Joseph et al. [13], who found that to get fresh air, reduce stress, and exercise were the dominant reasons why people visit parks. Our results also relate to those of Van Cauwenberg et al. [32] who noted that the closer non-retired individuals lived to a park, the higher the likelihood they would engage in recreational walking. Forty-four percent of park users engage in moderate to vigorous physical activity, not quite the majority as Joseph et al. [13] noted in their work, but substantially the same. Social reasons for visiting a park were not cited as highly as other reasons, and this disagrees with the work of Yung et al. [8], but their focus was on park visitation by the elderly, and this may account for the differences.

Not much previous work has focused on park visitation by young adults, and so it is difficult to compare the findings with those of others. Possibly, the closest work is that of Veitch et al. [21] who looked at park visitation by adolescents. They found the inclusion of slides, swings, and other amenities it the park was important in this context. The results presented here found little difference between the frequency of park visit behavior across all age groups, so nothing in particular was noted for the young adult age group, except in the case of monthly visits by the 18–24 age group, indicating they would be twice as likely to visit monthly as those aged over 50.

The perceived sound level and how the individuals value the park as a quiet area were not found to be significant predictors for park visit frequency, and this was somewhat unexpected. From the review of other work, such as that of Liu et al. [29], it was expected that these variables would be significant in influencing park visit frequency. The focus of Liu et al. [29] was on particular sounds as opposed to the sound level itself, and they noted that the more pleasing sounds of birdsong and trees rustling are being drowned out by traffic noise and other unwanted noise in parks. Traffic noise was found by them to negatively affect the park visiting experience. Gozalo et al. [30] also found an association between the absence of noise in urban green spaces and the reported satisfaction level of visits to them. Most of the respondents considered the sound levels to be low or adequate in the work presented here, with only 4% suggesting the parks were noisy or very noisy. This may explain why the sound level did not feature prominently in influencing park visit frequency.

A strength of the work presented here is the large number of participants in the survey, but a weakness is that the parks were only characterized by two variables, both of which focused on perception of the soundscape. Another limitation of the work is the focus on staff and students in a university in the survey, and this creates a bias in the results, the extent of which is difficult to quantify. A further limitation is that the results could not be followed up in post-survey interviews to explore whether the focus on park visit frequency as the dependent variable missed a potentially more nuanced influence of the park soundscape on the park visit experience.

The key findings from this work include that the proximity of people's residence to parks and why people visit (mainly to engage in physical activity) are more important than the characteristics of the individuals, as measured by age and gender, by how noisy the park is perceived, and whether the individuals value it as a quiet area. This suggests that the priority should be to include park space and open green areas in urban areas close to residential areas, to serve all age groups, even if the park space in terms of soundscape is not considered a quiet, tranquil space.

## 5. Conclusions

The research set out to explore the importance of a number of variables in park visit frequency. The variables were loosely grouped into variables that describe the individual, the reasons why they visit a park, and the perceived soundscape in the park. The findings suggest that the days of the week that individuals visit the park, the proximity of their residence to the park, and the reasons why they use the park are the strongest predictors of park visit frequency. Those variables are followed in importance by visiting the park with others, duration of the stay, age, and gender, in that order. The two variables defining the noise levels in the park and whether people considered the park valuable as a quiet area were not statistically significant. The contribution of these results, in terms of the importance of park availability for engagement in physical activity, suggest that the priority should be on the provision of park space close to residential areas, so that individuals can engage in activities such as walking and sports, and that the quality of that space, with respect to noise levels, is of secondary concern.

## Figures and Tables

**Table 1:** Variable definition and frequencies.

Variable Definition		N	%
Thinking of the park you visit the most, how often do you visit?	More than 3 times per week	108	12.5
	Weekly	239	27.6
	Monthly	280	32.4
	Less often than monthly ^r^	238	27.5
Are you male or female?	Female	538	62.2
	Male ^r^	327	37.8
Please specify your age group?	18–24	474	54.8
	25–34	134	15.5
	35–49	157	18.2
	>50 ^r^	100	11.6
Do you visit the park alone or with others?	Alone	260	30.1
	With others	481	55.6
	Both ^r^	124	14.3
How close do you live to your chosen park?	Within walking distance	492	56.9
	Within 10 min driving distance	176	20.3
	More than 10 min driving distance ^r^	197	22.8
Which of the following days of the week do you use this park?	Weekdays	206	23.8
	Any day	187	21.6
	Weekends ^r^	472	54.6
How long is the average duration of your stay in the park?	< 30 min	295	34.1
	1 h	443	51.2
	2 or more hours ^r^	127	14.7
What is your main reason for using this park?	Walking	293	33.9
	Jogging/Sport	89	10.3
	Relaxation	304	35.1
	Socialise ^r^	140	16.2
How would you describe the sound level in the park?	Very quiet	53	6.1
	Quiet	427	49.4
	Adequate	343	39.7
	Noisy or very noisy ^r^	42	4.9
Do you agree this park is valuable as a ‘quiet area’?	Strongly Agree	340	39.3
	Somewhat agree	368	42.5
	Neutral	106	12.3
	Somewhat or strongly Disagree ^r^	51	5.9
Total		865	100

Note: ^r^ refers to reference category, i.e., the category against which the other categories are referenced in the multinomial logistic regression (MNL) regression.

**Table 2:** Model fitting information and statistical significance of the independent variables.

Model Fitting Information				
**Model**	**Model Fitting Criteria** **-2 Log Likelihood**	**Likelihood Ratio Tests**		
		**Chi-Square**	**df**	**Sig.**
Intercept Only	2193.360	402.606	63	0.000
Final	1790.760			
Nagelkerke R^2^	0.4			
**Effect**	**Model Fitting Criteria** **-2 Log Likelihood of Reduced Model**	**Likelihood Ratio Tests**		
		**Chi-Square**	**df**	**Sig.**
Intercept	1790.6	0.00	0	
Gender	1799.6	8.84	3	0.03
Age	1812.2	21.42	9	0.01
Visit the park alone or with others	1823.8	33.02	6	0.00
Proximity of residence to park	1860.2	69.45	6	0.00
Days of the week on which visits to park take place	1923.5	132.70	6	0.00
Duration of stay	1815.5	24.75	6	0.00
Main reason for using the park	1833.6	42.79	9	0.00
Sound level in the park	1806.2	15.45	9	0.08
Agree or disagree that the park is valuable as a quiet area	1806.6	15.80	9	0.07

Note: df = degrees of freedom, Sig = statistical significance.

**Table 3:** Multinomial logistic regression parameter estimates.

Variables		> 3 times per week			Weekly			Monthly		
Frequency of park visits ^a^		*p*	OR	95% CI	*p*	OR	95% CI	*p*	OR	95% CI
Gender	Female	0.25	0.71	0.35, 1.29	**0.008**	**0.57**	**0.37, 0.86**	0.7	0.93	0.63, 1.37
	Male	-	-	-	-	-	-	-	-	-
Age	18–24	0.44	0.71	0.3, 1.68	0.4	1.33	0.69, 2.59	**0.02**	**2.1**	**1.1, 3.95**
	25–34	0.45	0.67	0.24, 1.89	0.27	1.55	0.72, 3.33	0.4	1.38	0.65, 2.89
	35–49	0.67	1.25	0.45, 3.4	0.1	1.87	0.88, 3.95	0.49	1.3	0.63, 2.69
	>50	-	-	-	-	-	-	-	-	-
Visit the park alone or with others	Alone	0.32	1.6	0.63, 4.0	0.65	0.84	0.4, 1.76	0.15	0.6	0.3, 1.2
	With others	**0.03**	**0.36**	**0.15, 0.89**	**0.001**	**0.31**	**0.16, 0.62**	**0.003**	**0.38**	**0.2, 0.7**
	Both	-	-	-	-	-	-	-	-	-
Proximity of residents to park	Within walking distance	**0.000**	**8.1**	**3.6, 18**	**0.000**	**7.5**	**4.3, 13.0**	**0.000**	**2.99**	**1.93, 4.65**
	Within 10 mins drive	**0.004**	**4.2**	**1.59, 11.1**	**0.000**	**3.66**	**2.0, 6.8**	**0.049**	**1.69**	**1.00, 2.86**
	More than 10 mins drive	-	-	-	-	-	-	-	-	-
Days of the week on which visits to the park take place	On weekdays	**0.000**	**9.0**	**3.68, 22.0**	**0.036**	**1.8**	**1.04, 3.12**	0.22	1.36	0.83, 2.22
	Any day	**0.000**	**45.2**	**19.1, 106.9**	**0.001**	**2.73**	**1.5, 4.98**	0.52	1.22	0.67, 2.22
	Weekends	-	-	-	-	-	-	-	-	-
Duration of stay	<30 mins	0.66	0.77	0.25, 2.42	**0.000**	**0.23**	**0.12, 0.47**	0.064	0.55	0.29, 1.04
	1 hour	0.77	0.85	0.29, 2.47	**0.09**	**0.6**	**0.34, 1.08**	0.79	0.93	0.53, 1.61
	2 or more hours	-	-	-	-	-	-	-	-	-
Main reason for using the park	Walking	**0.015**	**3.34**	**1.26, 8.84**	**0.003**	**2.58**	**1.39, 4.8**	0.063	1.69	0.97, 2.94
	Jogging/Sport	**0.047**	**3.1**	**1.02, 9.2**	**0.008**	**2.78**	**1.3, 5.9**	0.6	1.21	0.59, 2.5
	Relaxation	0.28	0.56	0.2, 1.58	0.98	0.99	0.54, 1.83	0.75	1.09	0.65, 1.83
Sound level in the park	Very quiet	0.24	0.37	0.07, 1.92	0.79	1.2	0.3, 4.97	0.3	0.5	0.14, 1.85
	Quiet	0.052	0.3	0.09, 1.0	0.99	0.99	0.31, 3.15	0.59	0.77	0.3, 1.97
	Adequate	0.054	0.31	0.1, 1.0	0.37	1.68	0.54, 5.18	0.8	1.13	0.45, 2.8
	Noisy or very noisy	-	-	-	-	-	-	-	-	-
Agree or disagree that the park is valuable as a quiet area	Strongly agree	0.62	1.39	0.38, 5.04	0.1	2.23	0.86, 5.84	0.1	2.05	0.87, 4.8
	Somewhat agree	0.83	0.88	0.26, 2.99	0.39	1.49	0.6, 3.7	0.24	1.63	0.73, 3.65
	Neutral	0.82	1.16	0.32, 4.27	0.59	0.76	0.27, 2.1	0.78	0.88	0.36, 2.15
	Somewhat/Strongly disagree	-	-	-	-	-	-	-	-	-

^a^ The reference category is: Less often than monthly. Bolded value: *p* (statistical significance) < 0.05, OR = odds ratio, 95% CI—OR 95% confidence interval.
